# Metal–Organic Skeleton-Derived W-Doped Ga_2_O_3_-NC Catalysts for Aerobic Oxidative Dehydrogenation of *N*-Heterocycles

**DOI:** 10.3390/ma17194804

**Published:** 2024-09-29

**Authors:** Fan Zhang, Qiwen Zhang, Feng Zhang, Xiaolin Luo, Wei Wang

**Affiliations:** Key Laboratory of Advanced Molecular Engineering Materials, College of Chemistry and Chemical Engineering, Baoji University of Arts and Sciences, Baoji 721013, China; sunnyzhangfan@163.com (F.Z.); qiwenzhang99@163.com (Q.Z.); jimmy0217@126.com (F.Z.); luoxl225@163.com (X.L.)

**Keywords:** MOF-derived catalysts, Ga_2_O_3_-NC, W doped, oxidative dehydrogenation, *N*-heterocycles

## Abstract

*N*-heterocycles with quinoline structures hold significant importance within the chemical and pharmaceutical industries. However, achieving their efficient transformations remains a vital yet challenging endeavor. Herein, a series of W-doped Ga_2_O_3_-NC catalysts were synthesized using a Ga-MOF-derived strategy through a simple solvothermal method, with a remarkably high activity and selectivity towards the oxidative dehydrogenation of *N*-heterocycles. Furthermore, the MOF-derived W-doped Ga_2_O_3_-NC catalysts exhibit remarkable substrate tolerance and recyclability. The outstanding catalytic activity was attributed to the robust synergistic interaction between the W species and the Ga_2_O_3_-NC carrier, which facilitates the activation of hydrogen atoms in the C-H and C=N bonds on both the oxygen molecule and the substrate to produce H_2_O_2_. Additionally, the solvent effect of methanol can significantly enhance dehydrogenation due to its strong ability to donate and accept protons of hydrogen bonding. The present work provides a new approach to MOF-derived non-precious metal catalysts for achieving the efficient oxidation dehydrogenation of *N*-heterocycles.

## 1. Introduction

The catalytic dehydrogenation of *N*-heterocycles holds significant importance in the field of synthetic chemistry due to the crucial role played by both unsaturated and saturated *N*-heterocycles as essential structural units in natural and pharmaceuticals products [1,2]. In comparison to the direct dehydrogenation route involving H_2_ removal, the oxidative dehydrogenation (ODH) pathway significantly reduces the required energy of the dehydrogenation thermodynamics with the help of reactive oxygen species, making ODH occur at low temperatures [3]. The utilization of heterogeneous noble metal catalysts, such as Pd [4], Pt [5,6], Ru [7], Rh [8], and Ir [9], is prevalent in the ODH of *N*-heterocycles due to their exceptional ability to activate O_2_. Despite their remarkable catalytic performance, the practical application of noble metal catalysts is greatly hindered by their exorbitant cost and limited availability [10,11]. Recently, certain non-noble metal catalysts have been employed for the selective oxidation dehydrogenation of *N*-heterocycles [12,13], which occasionally exhibit a commendable catalytic performance with the addition of additives [14]. A heterogeneous cobalt catalyst was prepared by loading cobalt oxide onto a support of nitrogen-doped carbon, facilitating the aerobic dehydrogenation of *N*-heterocycles at 60 °C using 1 equiv. of K_2_CO_3_ as an additive [15]. The development of an affordable and stable catalyst based on non-noble metals is highly desirable for the efficient chemical transformations of the ODH from saturated to unsaturated *N*-heterocycles.

Recently, metal–organic skeletons (MOFs) constructed from organic ligands and metal ions have been found to be outstanding precursors for the production of bifunctional or even multifunctional catalysts [16,17]. A number of publications have already reported the utilization of Ga for the synthesis of alkane *N*-heterocycle catalysts, where Ga is considered to function either as a promoter-element or as the active dehydrogenation component [18,19]. Carbon is a classically utilized material as a catalyst base for this reaction [13,20], especially for MOF-derived carbon–metal oxide catalysts. The “heteroatom doping” method is used to replace certain atoms of a carbon lattice by heteroatoms. Nitrogen doping is the most common type of doping and has been shown to be an effective method that has been applied to a variety of chemical reactions. Meanwhile, a number of papers have been published using Ga-MOF to efficiently catalyze N-heterocyclic reactions [21,22]; in these systems, gallium metal is considered as an accelerant or active dehydrogenation element. Usually, phosphotungstic acid hydrate is considered as a special dual-functional catalyst with complex, metal oxide, acidic, and redox properties [23,24]. Such a compound exhibits notable advantages including a high activity, excellent selectivity, and mild reaction conditions, rendering it to be extensively employed in diverse catalytic reactions [23]. However, it has the disadvantages of easy loss and recycling difficulties [20,24]. The establishment of a synergistic effect between two components holds significant importance for improving their oxidative activity [25,26]. Therefore, the incorporation of phosphotungstic acid as a W-doped precursor into GA-MOF-derived catalysts highlights a promising catalytic strategy for achieving efficient oxidative dehydrogenation.

Herein, a series of W-doped Ga_2_O_3_-NC samples with high activity, stability, and selectivity towards the ODH of *N*-heterocycles were synthesized using a Ga-MOF-derived strategy through a simple solvothermal method. The Ga-MOF-derived Ga_2_O_3_-NC framework with W doping introduces new catalytic active sites and significantly enhances specific surface area. The W/Ga_2_O_3_-NC catalysts were further characterized by a variety of techniques to disassociate the structure–activity relationships of high conversion efficiency for THQ oxidative dehydrogenation. Meanwhile, such catalysts can also be applied to the catalytic conversion of other *N*-heterocycles, such as isoquinoline, quinazoline, and quinoxaline derivatives. The present work provides a promising catalytic strategy of MOF-derived catalyst design for the efficient conversion of other *N*-heterocycles.

## 2. Materials and Methods

### 2.1. Materials

Phosphotungstic acid hydrate (H_3_O_40_PW_12_·xH_2_O, AR) was purchased from Aladdin. Gallium (III) nitrate hydrate (Ga(NO_3_)_3_·xH_2_O, 99.9%), N,N-dimethylformamide (DMF, 99.5%), and kalium carbonicum (K_2_CO_3_) were purchased from Macklin. 2-Aminoterephthalic acid, THQ (99.5%), and ultra-dry methyl alcohol (CH_3_OH, 99.9%) were purchased from Adamas. Oxygen (O_2_, 99.99%) was acquired from Xi’an-Teda-Cryogenic-Equipment Co., Ltd., Xi’an, China.

### 2.2. Catalyst Preparation

Typically, 1 mmol of Ga(NO_3_)_3_·xH_2_O, 3 mmol of 2-aminoterephthalic acid, and varying amounts (0, 0.05, 0.1, 0.2, 0.4, and 0.8 mmol) of H_3_O_40_PW_12_·xH_2_O were dissolved in 50 mL of DMF and subjected to stirring for 30 min. The formed solution was then transferred to a Teflon-lined autoclave (100 mL) and heated at 150 °C for 6 h. After the reaction, the resulting suspension was separated through centrifugation, washed three times with C_2_H_5_OH, and dried. The as-obtained powder was further heated for 4 h within a 600 °C tube furnace (5 °C·min^−1^ heating rate). These catalysts with different H_3_O_40_PW_12_·xH_2_O masses were labeled as Ga_2_O_3_-NC, W/Ga_2_O_3_-NC-1, W/Ga_2_O_3_-NC, W/Ga_2_O_3_-NC-2, W/Ga_2_O_3_-NC-3, W/Ga_2_O_3_-NC-4, and W/Ga_2_O_3_-NC-5, respectively. Moreover, other catalysts were also synthesized at different hydrothermal and calcined temperatures with 0.1 mmol H_3_O_40_PW_12_·xH_2_O. The products were labeled as PW-Ga@C-NH_2_(x-y), where “x” represents hydrothermal temperature and “y” represents calcined temperature.

### 2.3. Catalytic Reaction

The ODH of *N*-heterocycles was conducted in 16 mL Teflon-lined autoclaves. For a typical procedure, the ODH of N-heteroarenes was conducted by adding THQ (1 mmol), catalyst (20 mg), K_2_CO_3_ (1 mmol), and CH_3_OH (2 mL) into a sealed reactor. The reactor was then purged with O_2_ five times to remove any residual air. The reactor was pressured to 5 atm of O_2_, and was then heated to the desired temperature. Subsequently, the reactor was pressurized to 5 atm of O_2_ and was heated to begin the reaction at the required temperature. After the reaction, the reactor was gradually cooled to room-temperature and the gas was released slowly within the autoclave. The products were analyzed using GC-FID (Fuli, GC-9720) and GC-MS (Agilent 6890N-5975) following the centrifugal separation of the solid catalyst.

### 2.4. Characterization of Catalysts

The morphologies of the catalyst surface were observed using transmission electron microscopy (TEM) with a FEI TF20 microscope, and field emission scanning electron microscopy (SEM) using a Zeiss MERLIN instrument. X-ray powder diffraction (XRD) patterns were acquired using a D8 Advance Bruker instrument with 0.154 nm Cu-Kα radiation. An ASAP 2460 instrument was used to obtain the N_2_ adsorption–desorption isotherms at 77 K. The Brunauer–Emmett–Teller (BET) method was utilized for the analysis of specific surface area. X-ray photoelectron spectroscopy (XPS) was applied to analyze the chemical states of the as-obtained catalysts, and the C 1s (284.6 eV) of adventitious carbon served as a calibration reference for all the binding energies. The micro-Raman Thermo DXRxi spectrometer was employed to determine the Raman spectra of the catalysts.

## 3. Results and Discussion

### 3.1. Characterization

**Figure 1:**
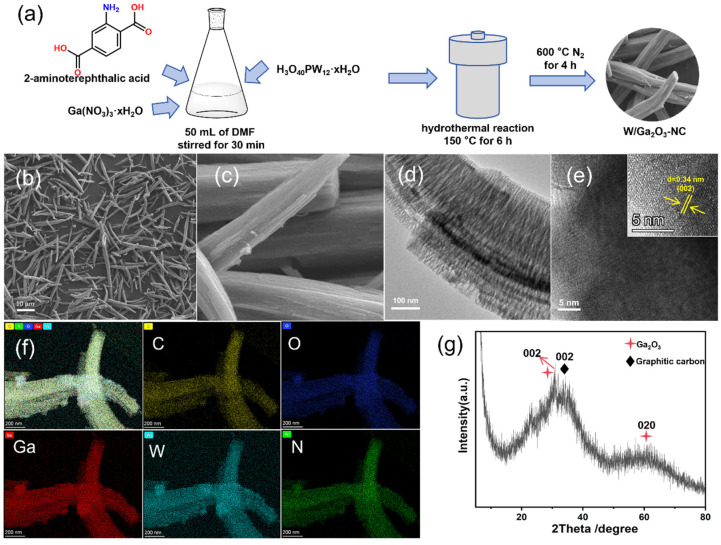
(**a**) Schematic illustration of the W/Ga_2_O_3_-NC preparation. (**b**,**c**) SEM images of W/Ga_2_O_3_-NC. (**d**,**e**) TEM and HRTEM images of the W/Ga_2_O_3_-NC catalyst. (**f**) EDX elemental mapping images. (**g**) XRD pattern of the W/Ga_2_O_3_-NC catalyst.

The preparation strategy of the W/Ga_2_O_3_-NC catalysts is illustrated in Figure 1a. The microstructures and morphologies of the MOF-derived W/Ga_2_O_3_-NC samples were observed using SEM, TEM, and elemental mapping. The SEM images of the W/Ga_2_O_3_-NC catalysts exhibit a kind of nanowire with a relatively uniform diameter (Figure 1b,c). Interestingly, the ports of the nanowires appear to be relatively regular and hexagonal (Figure S1). The TEM images clearly reveal the structure of the W/Ga_2_O_3_-NC catalyst, demonstrating that the metal particles are wrapped inside the carbon material, as depicted in Figure 1d and Figure S2. A further HRTEM image reveals a characteristic lattice with distances of 0.34 nm (Figure 1e), corresponding to the (002) facet of graphitic carbon [27], which is consistent with XRD results (Figure 1g). Furthermore, the XRD peaks at 31.7 and 60.9° can be assigned to the (002) and (020) facets of Ga_2_O_3_ (PDF#41-1103), implying the successful construction of a porous carbon-supported metal oxide catalyst derived from doped Ga-MOFs. Furthermore, the XRD pattern (Figure S3) also confirms the successful formation of Ga-MOF [28]. Consequently, Ga-MOF was calcined with phosphotungstic acid to form the desired W/Ga_2_O_3_-NC catalyst. The elemental mapping analysis further verifies the elements of C, N, O, Ga, and W in the catalyst (Figure 1f), demonstrating that the elements are evenly distributed in the entire framework. The above analysis demonstrates that Ga NPs are encapsulated in the carbon materials in the W/Ga_2_O_3_-NC sample.

**Figure 2:**
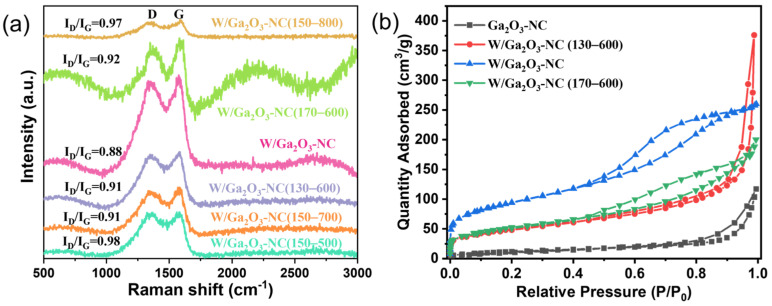
(**a**) Raman spectra and (**b**) N_2_ adsorption–desorption isotherms of the W/Ga_2_O_3_-NC catalysts.

The surface defects and the degree of carbon and nitrogen ordering in the W/Ga_2_O_3_-NC samples with different hydrothermal synthesis and calcination temperatures were investigated using Raman spectroscopy (Figure 2a). In the Raman spectra, two characteristic peaks located at 1356 and 1587 cm^−1^ can be clearly observed, corresponding to the D and G bands of the Raman spectrum that are characteristic of carbon materials, which can be assigned to the E_2g_ phonons of *sp*^2^ C atoms and the breathing mode of *k*-point phonons, respectively [29,30]. The D-band to G-band strength ratio (ID/IG) is an indicator of the degree of defects in carbon materials [30]. Obviously, the W/Ga_2_O_3_-NC sample exhibits significantly enhanced and narrower peaks for D and G bands (I_D_/I_G_ = 0.88), implying the highest degree order of the carbon matrix among the six samples. The structure of W/Ga_2_O_3_-NC (150–800) (I_D_/I_G_ = 0.97) exhibits a higher degree of disorder compared with that of W/Ga_2_O_3_-NC, which can be ascribed to the generation of more defects resulting from the removal of unstable atoms in the carbon matrix when overheated.

N_2_ physisorption was employed to study the pore structures of the Ga_2_O_3_-NC and W/Ga_2_O_3_-NC samples (Table S1). The introduction of W exhibits a significant impact on the BET surface area of the catalysts. The value of Ga_2_O_3_-NC was 50 m^2^/g, while the maximum (W/Ga_2_O_3_-NC) was increased markedly to 332 m^2^/g after the introduction of W. Meanwhile, the pore volume of W/Ga_2_O_3_-NC is 0.4 cm^3^/g, which is larger than that of other catalysts except for W/Ga_2_O_3_-NC (130-600). However, the W/Ga_2_O_3_-NC sample exhibits a significant advantage in specific surface area compared with W/Ga_2_O_3_-NC (130-600). Generally, the large pore volume and specific surface area can enhance the diffusion and adsorption capacity of small organic molecules [31,32]. This notion implies that the W/Ga_2_O_3_-NC samples may possess a higher catalytic activity compared with other catalysts. Figure 2b and S4 depict the N_2_ adsorption–desorption isotherms of the samples. The Ga_2_O_3_-NC and W/Ga_2_O_3_-NC catalysts display a type III isotherm, characterized by the absence of distinct knee points in the curve, indicating that these two materials have a larger pore diameter [30]. Additionally, other catalysts exhibit an isotherm of type IV, and display a hysteresis loop of type H4, demonstrating the involvement of a mesopore structure in these catalysts. Meanwhile, these curves show that the adsorption amount rises sharply when P/P_0_ was very low, which was related to a smaller pore diameter. These results can be further verified by measurements of pore size (Figures S5 and S6).

**Figure 3:**
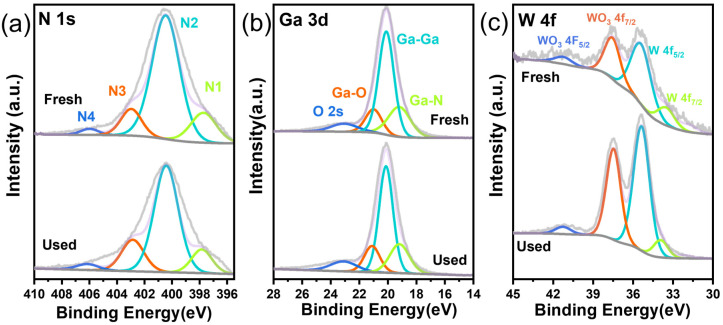
XPS spectra of the W/Ga_2_O_3_-NC catalyst. (**a**) N 1s, (**b**) Ga 3d, (**c**) W 4f.

XPS tests were employed to acquire the detailed chemical composition on the catalyst surfaces (Figure 3). N 1s spectra involved four fitted peaks—oxidized-type N species (406.2 eV, N4), graphitic-type N (403.6 eV, N3), pyrrolic-type N (400.5 eV, N2), and pyridinic-type N (398.1 eV, N1) [3,33]—while the Ga 3d spectra of W/Ga_2_O_3_-NC can be convoluted to four peaks for the values of binding energy at 19.2, 20.3, 21.2, and 23.6 eV attributing to Ga-Ga, Ga-N, Ga-O, and O 2s bonds, respectively [34,35,36]. The characteristic chemical states of W were exhibited, as evidenced by the presence of five peaks in the W 4f spectrum at 34.07, 35.69, 37.8, and 41.01 eV, corresponding to the transitions of 4f_7/2_, 4f_7/2,_ 4f_5/2_, and 5P_3/2_, respectively [37]. The XPS analysis results further confirmed the existence of Ga-N in the W/Ga_2_O_3_-NC catalyst. Additionally, the XPS analysis of the W/Ga_2_O_3_-NC catalysts before and after the reaction demonstrates their structural stability (Figure 3).

### 3.2. Catalytic Performance

The ODH of THQ towards quinoline was chosen as a reaction model to evaluate the performance of various catalysts, and the experimental results are collected in Table 1. The performance of sole H_3_O_40_PW_12_-xH_2_O and Ga_2_O_3_-NC catalysts was evaluated, achieving a THQ conversion of 54% and 48% with a quinoline selectivity of 91% and 85%, respectively (Table 1, entries 1 and 2). To compare with the H_3_O_40_PW_12_-xH_2_O and Ga_2_O_3_-NC catalysts, the W/Ga_2_O_3_-NC (x-y) samples (entries 3–12) exhibit a better oxidation dehydrogenation performance. Among these W-doped Ga_2_O_3_-NC catalysts, the W/Ga_2_O_3_-NC catalyst achieves the highest conversion of 99% with a selectivity of 89% (entry 4), which can be ascribed to its abundant pore channels and surfaces. Notably, the catalytic performance and economy of W/Ga_2_O_3_-NC were found to be notably superior compared with previously published catalytic systems (Table S2). Additionally, the results of the catalytic performances also found that a hydrothermal temperature of 150 °C and a calcined temperature of 600 °C are the optimal preparation conditions of the catalyst. Furthermore, an introduction amount of 0.1 mmol H_3_O_40_PW_12_·xH_2_O is found to be the optimal precursor quantity for the W source.

**Figure 4:**
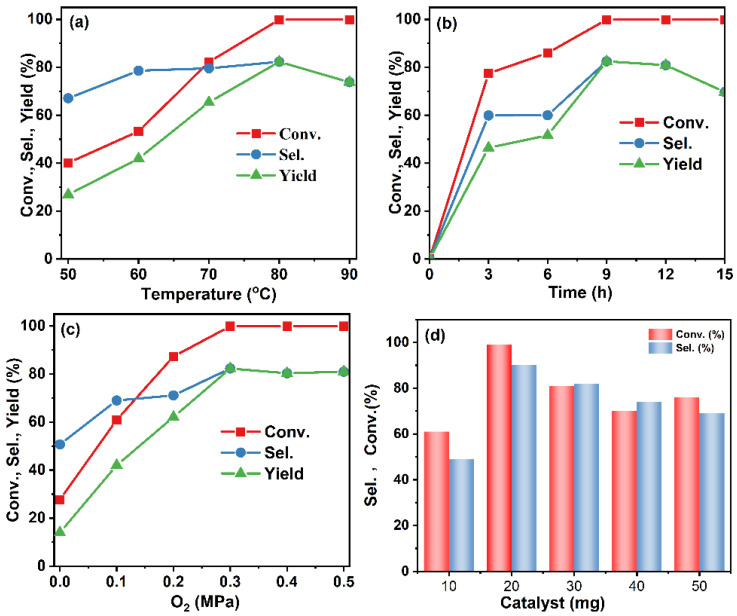
Impact of reaction conditions on the catalytic performance of W/Ga_2_O_3_-NC catalysts. (**a**) Reaction temperature, (**b**) reaction time, (**c**) oxygen pressure, and (**d**) catalyst dosage.

The W/Ga_2_O_3_-NC catalyst was subsequently employed to investigate the impact of each reaction factor on the catalytic activity and selectivity for the ODH of THQ (Figure 4). The conversion of THQ was enhanced by increasing the dehydrogenation temperature from 50 to 90 °C (Figure 4a), and the maximum conversion was achieved once the temperature reached 80 °C. However, the selectivity diminished as the temperature increased because of the excessive overoxidation at high temperatures [38]. During the experimental process, we detected overoxidized products, e.g., 3,4-dihydroquinolin-1(2*H*)-ol, etc., through confirmation with GC-MS analysis. To maximize quinoline yield, we selected an optimized temperature of 80 °C. The relationship between reaction time and catalytic performances is demonstrated in Figure 4b. The conversion exhibited a gradual increase with the extension of reaction time, while the selectivity for by-product formation decreased. The optimal reaction time was determined to be 9 h. The conversion of THQ was enhanced as the oxygen pressure increased from 0 to 0.4 MPa; when the oxygen pressure exceeds 0.4 MPa, the conversion of THQ no longer changes significantly (Figure 4c). Furthermore, Figure 4d reveals that the catalytic performance efficiency increases with an increase in catalyst dosage, as evidenced by the enhanced conversion and selectivity. However, at a catalyst amount of 20 mg, the catalytic activity reaches its optimum level. The reaction solvent was further screened, and the corresponding results are depicted in Figure S7. Methanol, as a strong polar solvent, exhibits the highest catalytic activity for the ODH of tetrahydroquinoline, with a conversion rate of 99% and an impressive yield of 88%. In general, the methanol solvent acts as a strong proton donor and acceptor for hydrogen bonds, which can effectively promote the transfer of protons during the dehydrogenation, thus promoting the overall process. Ethanol, on the other hand, displays secondary activity in the oxidative dehydrogenation of THQ. With 1,3,5-mesitylene and 1,4-dioxane as solvents, the yields were only 31% and 14%, respectively. Therefore, the optimal reaction conditions were determined as a temperature of 80 °C, a reaction time of 9 h, an oxygen pressure of 0.3 MPa, 20 mg of catalyst, and methanol as the reaction solvent.

**Figure 5:**
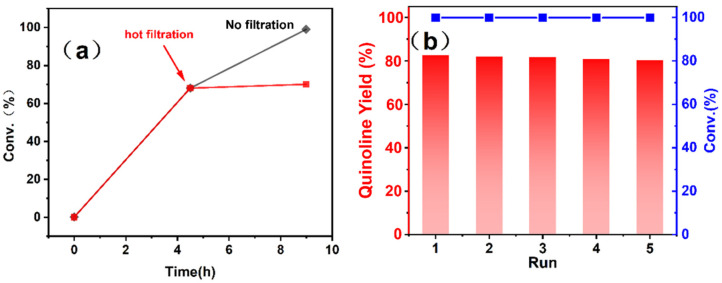
Stability (**a**) and cycling (**b**) tests of catalysts in the oxidative dehydrogenation reaction of tetrahydroquinoline over a W/Ga_2_O_3_-NC catalyst.

Under the optimal reaction conditions, thermal filtration tests were conducted (Figure 5a). The specific operation involved rapid catalyst removal through filtration and subsequent analysis by gas chromatography at a reaction time of 4.5 h. After a reaction time of 4.5 h, no further increase in the conversion rate of the test reaction was observed, indicating the non-homogeneous participation of the catalyst in the reaction. To assess the stability of the W/Ga_2_O_3_-NC catalyst, we conducted a study on its reusability in the ODH of THQ under optimized conditions, and the results are presented in Figure 5b. The results indicate that the W/Ga_2_O_3_-NC catalyst exhibits excellent reusability, maintaining its activity without an obvious loss with at least five consecutive uses. SEM and TEM images (Figure S8) of the W/Ga_2_O_3_-NC catalyst after the reaction demonstrate no discernible changes in the morphology and roughness of its surface. Meanwhile, XPS analysis of the W/Ga_2_O_3_-NC catalyst before and after the dehydrogenation also confirms its structural stability (Figure 3), which implies its potential as an industrial catalyst.

### 3.3. Substrate Expansion

To assess the universality of the W/Ga_2_O_3_-NC catalyst, we conducted a comprehensive investigation into its applicability in the ODH of different *N*-heterocyclic substrates (Table 2). The dehydrogenation of quinolines substituted with electron-withdrawing and electron-donating groups was successfully achieved. The substrate at the 6-position of a methyl group of THQ achieved an almost complete conversion (>99%) with a selectivity of 91% towards the desired product (Table 2, entry 1). Moreover, the substrate at the 2-position of the methyl group of THQ exhibited an excellent conversion (>99%) and a medium selectivity (58%) towards 2-methylquinoline in the titled reaction (entry 2). The substrate was completely converted (>99%) by 6-Hydroxy-1,2,3,4-tetrahydroquinoline with a selectivity of only 65% towards the corresponding product (entry 3), while 7-nitro-1,2,3,4-tetrahydroquinoline achieved a conversion of 87% with a high selectivity of 99% after 15 h of reaction (entry 4). Obviously, the as-developed catalytic system also exhibits a notable capability in facilitating the ODH of indole and isoquinoline, resulting in corresponding products with remarkable yields of 99% and 85%, respectively (entries 5 and 6). More markedly, it has an extremely efficient catalytic performance for the ODH of indole, demonstrating a remarkable universality of the present protocol. Additionally, a moderate yield of 51% was obtained for the conversion of 2-methylindole towards 2-methyl-1*H*-indole, while the reactions of 7-chloroindoline and 5-bromoindoline resulted in yields of 89% for the formation of 7-chloro-1*H*-indole and only 23% for the production of 5-bromo-1*H*-indole. These results suggest that the presence of the electron-withdrawing and steric hindrance groups hindered the reaction (entries 7–9).

### 3.4. Catalytic Mechanism of THQ Dehydrogenation

**Figure 6:**
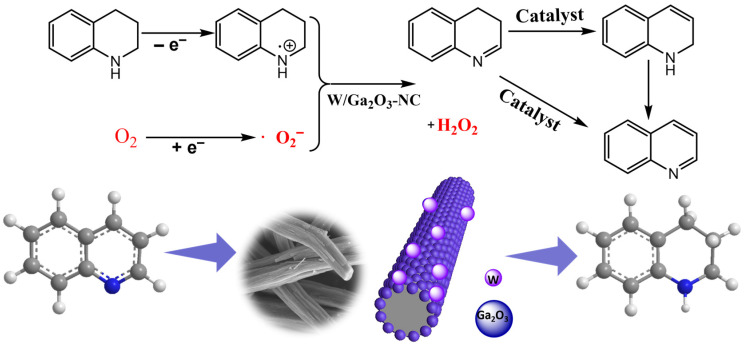
A possible mechanism for the aerobic oxidative dehydrogenation of THQ over a W/Ga_2_O_3_-NC catalyst.

The outstanding dehydrogenation performance of the W/Ga_2_O_3_-NC catalyst may be ascribed to the incorporation of W-doping in Ga-MOF-derived carbon and nitrogen materials, resulting in the formation of defects that serve as active sites for the adsorption and reduction of O_2_. Based on the experimental results and corresponding reports [4,38,39,40], a possible mechanism for the aerobic oxidative dehydrogenation of THQ was proposed over a W/Ga_2_O_3_-NC catalyst (Figure 6). The synergistic effect between the nitrogen–carbon-coated catalytic active substance W species and Ga_2_O_3_-NC can adsorb O_2_ from the surrounding air, enabling electrons to reduce O_2_ to ·O_2_^−^, thus promoting the oxidative dehydrogenation of THQ. Notably, the addition of benzoquinone (an ·O_2_^−^ trapping agent) dramatically decreased the catalytic activity and selectivity for the oxidative dehydrogenation of *N*-Heterocycles (Figure S9), confirming the crucial role played by ·O_2_^−^ species in this process. Meanwhile, in the N-coordination environment, electrons adsorbed on the surface of metal nanorods undergo a process of electron transfer to nitrogen atoms [41,42], which is conducive to the generation of electron transfer ·O_2_^−^, while methanol, as a solvent, can serve as an effective proton donor for the transformation of ·O_2_^−^ into the potent oxidizing substance HO_2_^−^ species, which further activates the N-H bond on THQ and releases hydrogen in the adjacent C-H bond to form imine intermediates. Meanwhile, the activated hydrogen is released again and combines with HO_2_^−^ to form H_2_O_2_. This leads to the further oxidation of imine intermediates to obtain target quinoline products.

## 4. Conclusions

In summary, a series of W-doped Ga_2_O_3_-NC catalysts were synthesized by a Ga-MOF-derived strategy through a simple solvothermal method. The W/Ga_2_O_3_-NC catalyst achieved a high activity and selectivity towards the oxidative dehydrogenation of *N*-heterocycles. The Ga-MOF-derived Ga_2_O_3_-NC framework with W doping introduces a new catalytically active species and increases the specific surface area. Such heterogeneous catalysts exhibit remarkable substrate tolerance, catalytic activity, and recyclability. Furthermore, it was observed that the outstanding catalytic performance was ascribed to the strong synergistic interaction between the W species and the Ga_2_O_3_-NC carrier, which facilitates the activation of hydrogen atoms in C-H and C=N bonds on both the oxygen molecule and substrate to produce H_2_O_2_. The present work provides a new approach for constructing redox catalysts, and presents a technical and feasible method of employing non-precious metal catalysts as substitutes for conventional precious metal catalysts in the oxidation dehydrogenation of *N*-heterocycles.

## Figures and Tables

**Table 1:** Catalytic activity and selectivity of the ODH of THQ over various catalysts *^a^*.

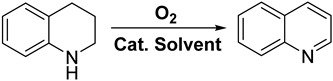				
Entry	Catalyst	Conv. *^b^* (%)	Sel. *^b^* (%)	Yield (%)
1	H_3_O_40_PW_12_-xH_2_O	54	91	49
2	Ga_2_O_3_-NC	48	85	41
3	W/Ga_2_O_3_-NC (130-600)	98	80	78
4	W/Ga_2_O_3_-NC *^c^*	99	89	88
5	W/Ga_2_O_3_-NC (170-600)	94	71	67
6	W/Ga_2_O_3_-NC (150-500)	97	60	58
7	W/Ga_2_O_3_-NC (150-700)	98	79	77
8	W/Ga_2_O_3_-NC (150-800)	70	70	49
9 *^c^*	W/Ga_2_O_3_-NC-1	83	82	68
10 *^c^*	W/Ga_2_O_3_-NC-2	88	80	70
11 *^c^*	W/Ga_2_O_3_-NC-3	97	79	77
12 *^c^*	W/Ga_2_O_3_-NC-4	93	73	68

*^a^* Reaction conditions: THQ 1 mmol, catalyst 20 mg, methanol 2 mL, K_2_CO_3_ 1 mmol, O_2_ 5 atm, 80 °C, 9 h. *^b^* Determined by GC with toluene as internal standard, the by-products were the overoxidized products including 3,4-dihydroquinolin-1(2*H*)-ol, etc. *^c^* W/Ga_2_O_3_-NC (150–600).

**Table 2:** The universality investigation for the ODH of different substrates over a W/Ga_2_O_3_-NC catalyst *^a^*.

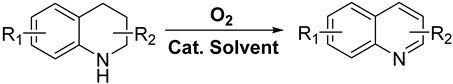						
Entry	Substrates	Products	T (h)	Conv. *^b^* (%)	Sel. *^b^* (%)	Yield *^b^* (%)
1	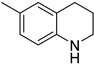	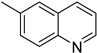	12	>99	91	91
2	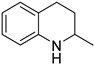	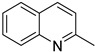	12	>99	58	58
3	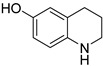	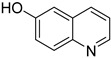	15	>99	65	65
4	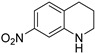	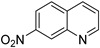	15	87	>99	87
5	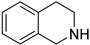	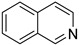	12	85	>99	85
6	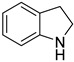	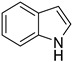	12	>99	100	>99
7	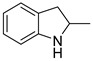	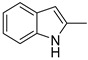	12	51	100	51
8	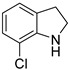	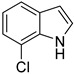	12	89	100	89
9	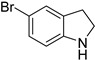	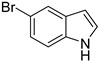	12	23	100	23

*^a^* Reaction conditions: substrate 0.1 mmol, P/Ga@C-NH_2_ 20 mg, CH_3_OH 2 mL, O_2_ 0.4 MPa, K_2_CO_3_ 1mmol. *^b^* Determined by GC with toluene as an internal standard.

## Data Availability

The original contributions presented in the study are included in the article/Supplementary Materials, further inquiries can be directed to the corresponding author.

## Supplementary Materials

The following supporting information can be downloaded at: https://www.mdpi.com/article/10.3390/ma17194804/s1, Figure S1: SEM images of W/Ga_2_O_3_-NC; Figure S2: TEM images of W/Ga_2_O_3_-NC; Figure S3: XRD pattern of the Ga-MOF sample; Figure S4: N_2_ adsorption–desorption isotherms for different hydrothermal reaction temperatures; Figure S5: BJH pore size distribution of Ga_2_O_3_-NC and W/Ga_2_O_3_-NC for different hydrothermal reaction temperatures; Figure S6: BJH pore size distribution of Ga_2_O_3_-NC and W/Ga_2_O_3_-NC for different calcination temperatures; Figure S7: Effect of solvent type on the catalytic process of the oxidative dehydrogenation of THQ; Figure S8: SEM (a) and TEM images of W/Ga_2_O_3_-NC after reaction; Figure S9: Radical scavenging experiments for the oxidative dehydrogenation over a THQ W/Ga_2_O_3_-NC catalyst; Table S1: BET surface area, pore size, and pore volume of as-prepared catalysts; Table S2: Comparison of dehydrogenation performances for *N*-heterocycles of reported catalysts.
