# Supplementary material for: Metal–Organic Skeleton-Derived W-Doped Ga2O3-NC Catalysts for Aerobic Oxidative Dehydrogenation of N-Heterocycles
Source: Materials (Basel). 2024 Sep 29;17(19):4804. doi: 10.3390/ma17194804 (PMC11477574; doi:10.3390/ma17194804)
Supplement: Supplementary file 1 [file materials-17-04804-s001.zip › materials-3193609-supplementary.pdf]

Supplementary Material

Metal–Organic Skeleton-Derived W-Doped Ga_2O_3-NC Catalysts for Aerobic Oxidative Dehydrogenation of *N*-Heterocycles

Fan Zhang, Qiwen Zhang, Feng Zhang, Xiaolin Luo and Wei Wang *

Key Laboratory of Advanced Molecular Engineering Materials, College of Chemistry and Chemical Engineering, Baoji University of Arts and Sciences, Baoji 721013, China; sunnyzhangfan@163.com (F.Z.); qiwenzhang99@163.com (Q.Z.); jimmy0217@126.com (F.Z.); luoxl225@163.com (X.L.)

* Correspondence: weiwang204@163.com; Tel.: +86-(0)917-3566589

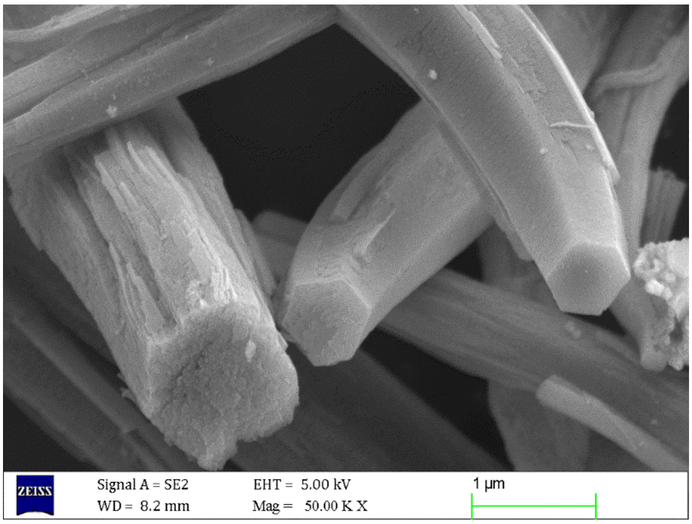

Figure S1. SEM images of W/Ga_2O_3-NC.

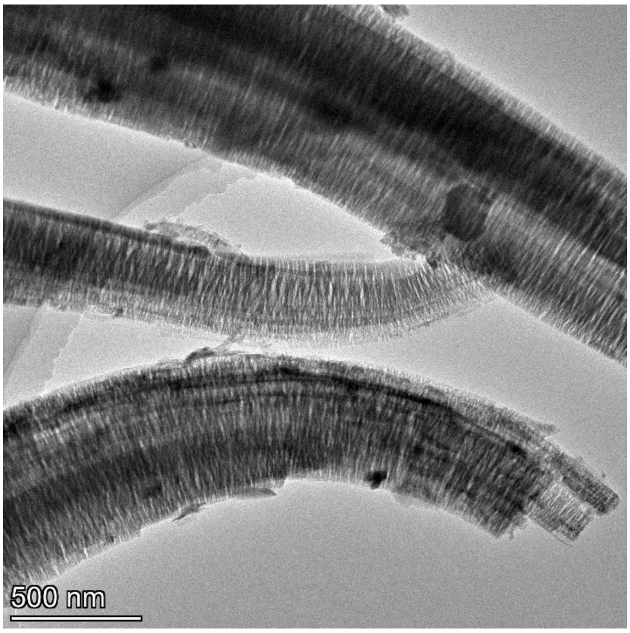

Figure S2. TEM images of W/Ga_2O_3-NC.

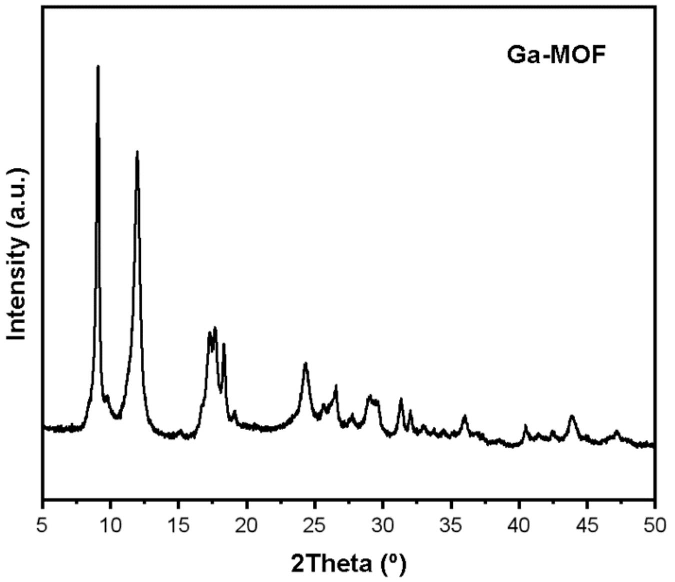

Figure S3. XRD pattern of Ga-MOF sample.

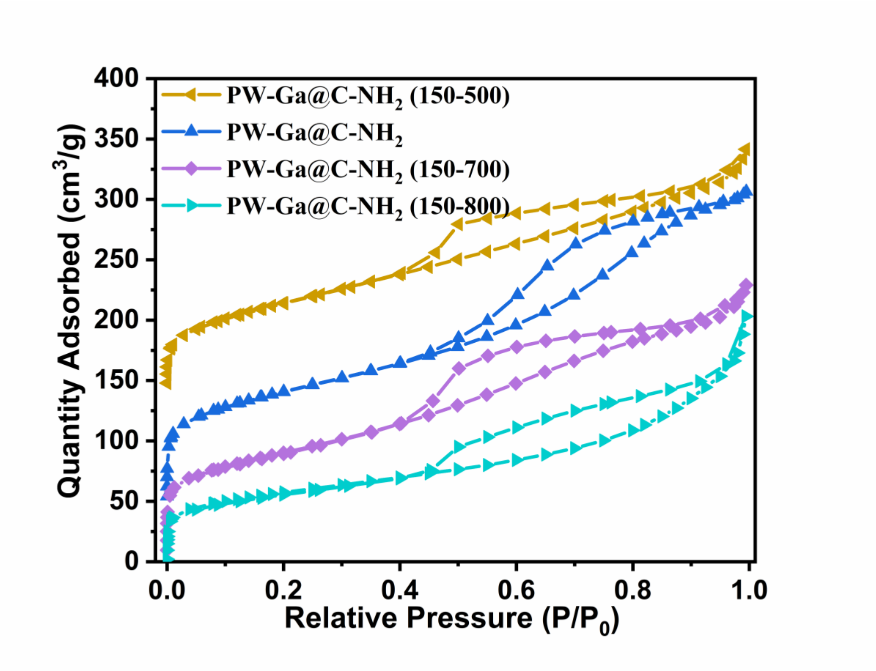

Figure S4. N_2-adsorption-desorption isotherms for different hydrothermal reaction temperature.

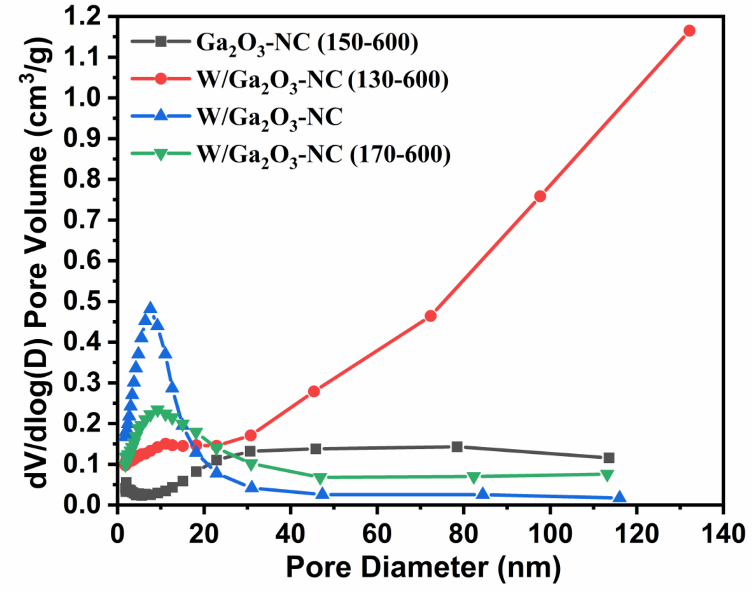

Figure S5. BJH pore size distribution of Ga2O3-NC and W/Ga2O3-NC for different hydrothermal reaction temperature.

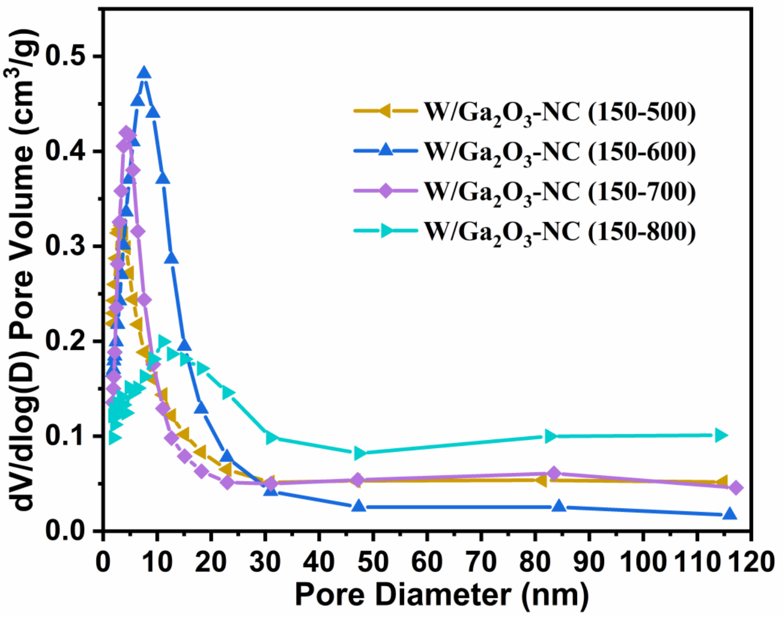

Figure S6. BJH pore size distribution of Ga2O3-NC and W/Ga2O3-NC for different calcination temperature.

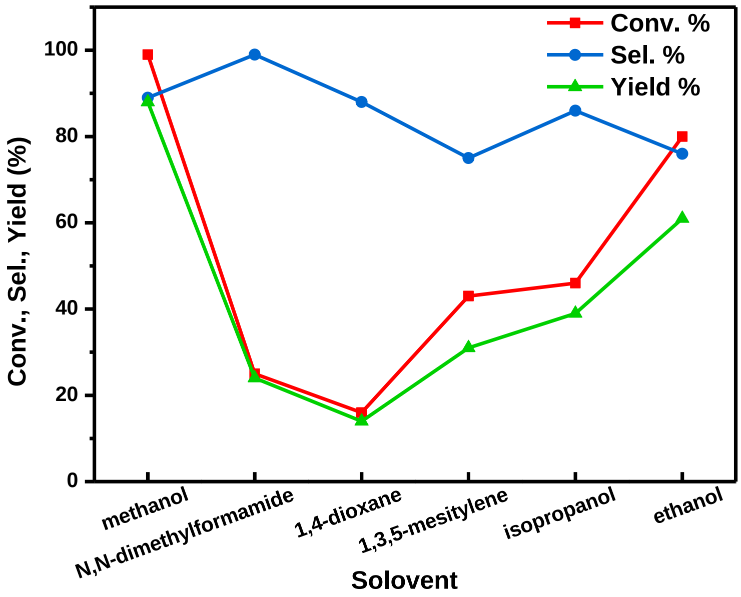

Figure S7. Effect of solvent type on the catalytic process of oxidative dehydrogenation of THQ.

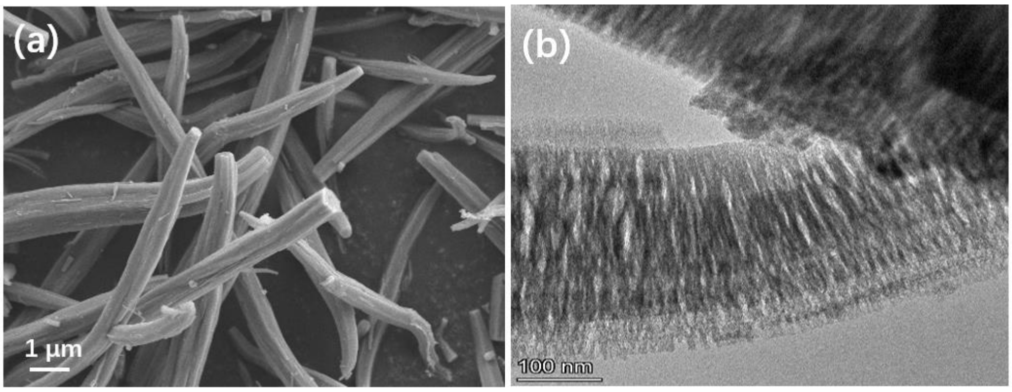

Figure S8. SEM (a) and TEM images of W/Ga_2O_3-NC after reaction.

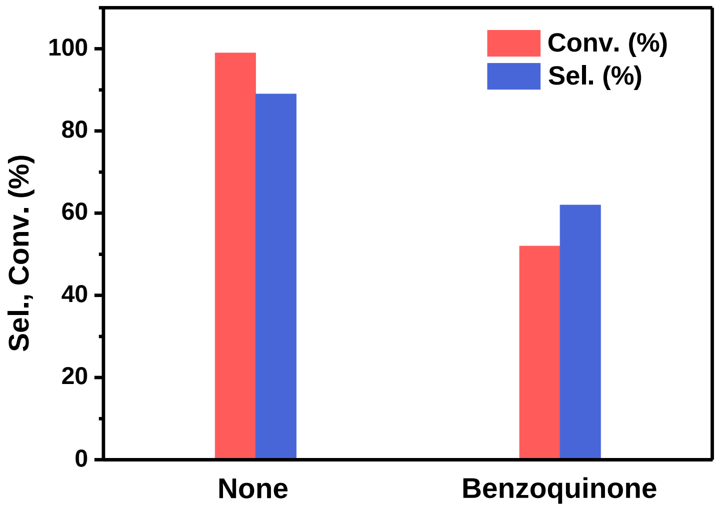

Figure S9. Radical scavenging experiments for the oxidative dehydrogenation over THQ W/Ga_2O_3-NC catalyst.

Table S1. BET surface area, pore size, and pore volume of as-prepared catalysts.

Entry	Catalysts	S_{BET} (m^2/g)	D_{pore} (nm)	V_{pore} (cm^3/g)
1	W/Ga_2O_3-NC (150-500)	260	4.5	0.3
2	W/Ga_2O_3-NC[a]	332	4.8	0.4
3	W/Ga_2O_3-NC (150-700)	305	4.5	0.3
4	W/Ga_2O_3-NC (150-800)	218	5.8	0.3
5	W/Ga_2O_3-NC (130-600)	172	13.2	0.6
6	W/Ga_2O_3-NC (170-600)	182	6.6	0.3
7	Ga_2O_3-NC	50	14.3	0.2

[a]W/Ga_2O_3-NC (150-600)

Table S2. Comparison of dehydrogenation performances for N-heterocycles of reported catalysts.

Catalyst	Time (h)	T (°C)	Gas	Yield (%)	Ref.
RhCNT/TBC	12	room temperature	Air	82.0	[1]
Ni_2Mn-LDH	12	100	O_2	85.0	[2]
FeO_x@NGr−C	12	100	Air	83.0	[3]
Fe-L1@EGO-900	18	145	Ar	92.0	[4]
Ru_3O_2/rGO	18	room temperature	O_2	86.0	[5]
PdNPs/SBA-15	23	130	Air	98.0	[6]
Co@NGS-800[a]	6	80	O_2	82.0	[7]
Co_3O_4/AlN	12	120	O_2	88.0	[8]
**W/Ga_2O_3-NC**	**9**	**80**	**O_2**	**88.0**	**This work**

[a]Catalyst assistance: K_2CO_3

References

[1] D.V. Jawale, E. Gravel, N. Shah, V. Dauvois, H. Li, I.N.N. Namboothiri, E. Doris, Cooperative dehydrogenation of N-heterocycles using a carbon nanotube-rhodium nanohybrid, Chem. Eur. J., 21 (2015) 7039-7042.

[2] W. Zhou, Q. Tao, F.a. Sun, X. Cao, J. Qian, J. Xu, M. He, Q. Chen, J. Xiao, Additive-free aerobic oxidative dehydrogenation of N-heterocycles under catalysis by NiMn layered hydroxide compounds, J. Catal., 361 (2018) 1-11.

[3] X. Cui, Y. Li, S. Bachmann, M. Scalone, A.-E. Surkus, K. Junge, C. Topf, M. Beller, Synthesis and characterization of iron–nitrogen-doped graphene/core–shell catalysts: Efficient oxidative dehydrogenation of N-heterocycles, J. Am. Chem. Soc., 137 (2015) 10652-10658.

[4] G. Jaiswal, V.G. Landge, D. Jagadeesan, E. Balaraman, Iron-based nanocatalyst for the acceptorless dehydrogenation reactions, Nat. Commun., 8 (2017) 2147.

[5] X. Li, N. Guo, Z. Chen, X. Zhou, X. Zhao, Y. Du, L. Ma, Y. Fang, H. Xu, H. Yang, W. Yu, S. Lu, M. Tian, Q. He, K.P. Loh, S. Xi, C. Zhang, J. Lu, Atomically precise single metal oxide cluster catalyst with oxygen-controlled Aactivity, Adv. Fun. Mater., 32 (2022).

[6] C. Deraedt, R. Ye, W.T. Ralston, F.D. Toste, G.A. Somorjai, Dendrimer-dtabilized metal nanoparticles as efficient catalysts for reversible dehydrogenation/hydrogenation of N-heterocycles, J. Am. Chem. Soc., 139 (2017) 18084-18092.

[7] J. Li, G. Liu, X. Long, G. Gao, J. Wu, F. Li, Different active sites in a bifunctional Co@N-doped graphene shells based catalyst for the oxidative dehydrogenation and hydrogenation reactions, J. Catal., 355 (2017) 53-62.

[8] Z.-H. He, Y.-C. Sun, K. Wang, Z.-Y. Wang, P.-P. Guo, C.-S. Jiang, M.-Q. Yao, Z.-H. Li, Z.-T. Liu, Reversible aerobic oxidative dehydrogenation/hydrogenation of N-heterocycles over AlN supported redox cobalt catalysts, Mol. Catal., 496 (2020) 111192.
